# Lessons learned from a multi-site collaborative working toward a digital health use screening tool

**DOI:** 10.3389/fpubh.2024.1421129

**Published:** 2024-07-08

**Authors:** Ann M. Hernandez, Elaine C. Khoong, Neytali Kanwar, Naomi Lopez-Solano, Jorge A. Rodriguez, Emilia De Marchis, Oanh Kieu Nguyen, Alejandra Casillas

**Affiliations:** ^1^Department of Family Medicine, David Geffen School of Medicine at UCLA, Los Angeles, CA, United States; ^2^Division of General Internal Medicine at Zuckerberg San Francisco General Hospital, Department of Medicine, University of California, San Francisco, San Francisco, CA, United States; ^3^UCSF Action Research Center (ARC) for Health Equity, University of California, San Francisco, San Francisco, CA, United States; ^4^School of Medicine, University of California, San Francisco, San Francisco, CA, United States; ^5^Division of Hospital Medicine at Zuckerberg San Francisco General Hospital, Department of Medicine, University of California, San Francisco, San Francisco, CA, United States; ^6^Division of General Internal Medicine and Primary Care, Brigham and Women’s Hospital, Boston, MA, United States; ^**7**^Harvard Medical School, Boston, MA, United States; ^8^Department of Family and Community Medicine, University of California, San Francisco, San Francisco, CA, United States; ^9^Chan Zuckerberg Biohub, San Francisco, CA, United States; ^10^Division of General Internal Medicine and Health Services Research, Department of Medicine, David Geffen School of Medicine at UCLA, Los Angeles, CA, United States

**Keywords:** digital health technology (DHT), health equity (MeSH), social determinants of health, digital health (eHealth), digital divide (DD)

## Abstract

Digital health has the potential to expand health care and improve outcomes for patients—particularly for those with challenges to accessing in-person care. The acceleration of digital health (and particularly telemedicine) prompted by the Coronavirus-19 (COVID-19) pandemic facilitated continuity of care in some settings but left many health systems ill-prepared to address digital uptake among patients from underserved backgrounds, who already experience health disparities. As use of digital health grows and the digital divide threatens to widen, healthcare systems must develop approaches to evaluate patients’ needs for digital health inclusion, and consequentially equip patients with the resources needed to access the benefits of digital health. However, this is particularly challenging given the absence of any standardized, validated multilingual screening instrument to assess patients’ readiness for digital healthcare that is feasible to administer in already under-resourced health systems. This perspective is structured as follows: (1) the need for digital health exclusion risk screening, (2) our convening as a group of stakeholders, (3) our review of the known digital health screening tools and our assessment, (4) formative work with patients regarding their perceptions on language and concepts in the digital health screening tools, and (5) conclusion with recommendations for digital health advocates generated by this collaborative of digital health researchers and operations leaders. There is a need to develop a brief, effective tool to screen for digital health use that can be widely implemented in diverse populations. We include lessons learned from our experiences in developing and testing risk of digital health exclusion screening questions in our respective health systems (e.g., patient perception of questions and response options). Because we recognize that health systems across the country may be facing similar challenges and questions, this perspective aims to inform ongoing efforts in developing health system digital exclusion screening tools and advocate for their role in advancing digital health equity.

## 1 Background- *the digital divide demands a roadmap for digital health exclusion screening as a distinct social driver of health*

Since the implementation of the 2009 Health Information Technology for Economic and Clinical Health (HITECH) Act, health systems have integrated digital health as part of healthcare delivery— primarily via patient portals and telemedicine (e.g., remote clinical visits). Healthcare systems have looked to leverage digital tools to improve health outcomes, mitigate disparities, and enhance patient access and engagement (1–3). These digital tools also increased in response to the COVID-19 public health emergency (4). However, while these hold great potential, there are continued and snowballing inequities (5–9). Such multilevel digital health disparities limit the potential benefits of digital health, and more so, worsen inequities for patients from underserved backgrounds (racial or ethnic minority, limited English proficient (LEP), rural, older, or low socioeconomic status) who are *already* at baseline risk for worse health outcomes. This “digital divide” is partly characterized by limited digital literacy and experience, lower quality broadband or cellular service, and lack of reliable access to Internet-connected personal devices. Technology-specific challenges, including usability and English-only language of digital tool platforms, along with health system factors (e.g., lack of patient-centered education, limited staff trained to mitigate digital inclusion barriers), also contribute to these gaps. Because of these reasons, underserved patient populations, like those mentioned above, have faced barriers to accessing digital tools that can improve their health care (6–12). The evidence highlights the profound impact of the digital divide and the urgent need to address digital inclusion.

National Digital Inclusion Alliance (NDIA) defines digital equity as the “conditions in which every individual and community possesses the necessary information technology capacity to engage fully in society, democracy, and the economy,” (13) and digital inclusion as “efforts aimed at guaranteeing that diverse populations have access to and utilize information and digital technologies;” (13) NDIA outlines 5 pillars for digital inclusion: *affordability, robust broadband Internet service, Internet-enabled devices that meet user needs, access to digital literacy training, and quality technical support* (13). Advancing digital inclusion requires intentional investment in these pillars which address the historical, institutional, and structural barriers to accessing and utilizing technology. This should include *screening* for risk of digital health exclusion, much like we do now in health care systems for other social drivers of health— such as with food insecurity and housing.

Like other social drivers of health, digital inclusion intersects with educational opportunities, employment, social connection, information networks, and housing (14, 15). Although healthcare systems are expanding initiatives to address social needs (16), significant gaps remain in specifically addressing digital inclusion, especially among those populations that are at greatest risk of facing barriers (11, 12, 14, 17–19). A large challenge to screening is the limited formative research and lack of validated, evidence-based screening tools evaluated in diverse populations. Current approaches to screening for digital health use are not standardized and miss key pillars of digital inclusion. Furthermore, digital inclusion is not a core domain for social risk screening among growing national quality standards, most notably, not even for the Centers for Medicare and Medicaid Services (16, 20, 21).

**Figure 1:**
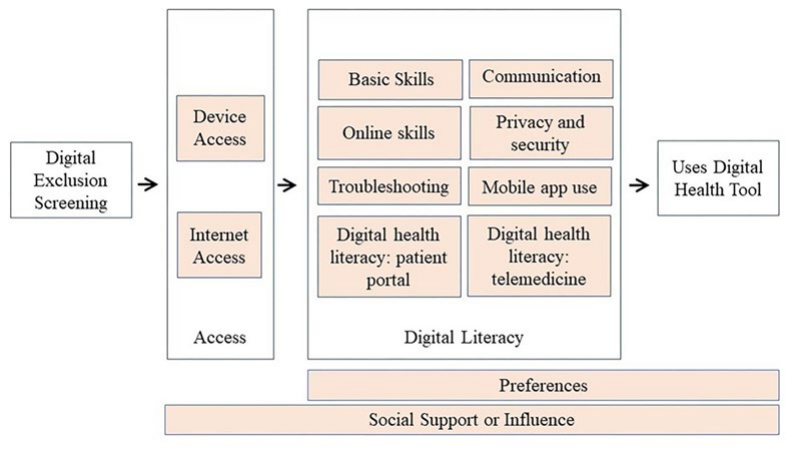
Conceptual framework for use of digital health tools. We conceptualize use of a digital health tool as requiring device and internet access, digital literacy, and preference for using the tool. Social support may impact any of these three constructs. Digital exclusion screening should focus on access and digital literacy.

This endeavor starts with a novel conceptual framework for the use of digital health tools (Figure 1) that reflects how we conceptualize the essential components required for use of digital health tools. Starting with appropriate Internet-connected device, broadband access (including home-based) and digital literacy, an individual’s preferences may result in limited use of a digital health tool. Social support or influence can impact device and Internet access, digital literacy, and/or preferences.

It is important to separately assess device access, Internet access, and digital literacy skills; the strategies to address each are different. For example, a patient who lacks access to low-cost Internet needs a different referral than the patient who does not have the digital literacy to register for their patient portal. And while preferences play a role in the *actual* use/uptake of a digital tool, screening for gaps is an initial and different goal. Similar to how one may screen for access to adequate food but not focus on preferences for uptake of those food resources.

In this perspective, we present our efforts in developing a screening tool to assess the risk of digital health exclusion across sites and populations. We will describe the following processes and experiences: (1) how our collaborative came together and met (actively engaged in initiatives and research in digital health equity), (2) our collective review of known screening tools and assessments for the risk of digital health exclusion (informed by the known literature around digital exclusion albeit limited), and (3) formative work with stakeholders on screening tools’ language and concepts (patient interviews and key informant conversations about screening questions). We conclude with recommendations based on this preliminary work – some common across sites and some site-specific – combined with our professional and personal experiences as healthcare providers, community partners, and digital health equity advocates in academic medical centers and county health systems.

## 2 Developing a multisite collaborative- *need to develop screening tools for the risk of digital health exclusion*

Our collaborative is composed of health services researchers, operations leaders, and medical trainees who have conducted research and implementation in digital health over the last decade within county health systems, community health centers, and academic medical centers. Our collaboration originated through shared projects which led to discussions regarding the need for digital health screening tools in our respective health settings and systems. We met virtually between September 2022 through March 2023 (participants: AC, ED, AH, EK, ON, JR). During these monthly sessions, we focused on identifying risk of digital health exclusion screening tools and/or questions from our own reviews of the literature and what had been used *ad hoc* after the start of the COVID-19 pandemic. We reviewed the complexities of screening for risk of digital health exclusion within our respective organizational contexts. Discussions encompassed the selection of existing questions that would comprehensively address the domains of digital exclusion, challenges faced by patients with limited English proficiency, considerations for digital exclusion screening in the inpatient versus outpatient settings, and the purpose of screening efforts (e.g., increasing video adoption versus increasing portal adoption versus digital inclusion broadly). Our conversations further delved into logistical considerations of written versus oral, self-administered versus proctored surveys, as well as the nuanced phrasing of questions about technology and health. Through this process, we identified common challenges which were further enriched by interviews with patients, and discussions with health system stakeholders.

## 3 The state of digital health screening- *identifying common challenges and persistent gaps*

Our collaborative identified over 20 questionnaires, only a handful published, from the literature, our own health systems, and among colleagues working in digital health. Some of the more common tools included: the eHealth Literacy Scale (eHEALS) (20), the Digital Health Care Literacy Scale (DHLS) (21), the Veteran’s Health Administration Assessing Circumstances and Offering Resources for Needs screening tool (ACORN) (22), the Mobile Device Proficiency Questionnaire (MDPQ) (23), as well as questions from the Health Information National Trends Survey (HINTS) (24) and the American Community Survey (ACS) (25).

The eHEALS consists of 8 questions and assesses respondents’ perceived skills and comfort with identifying and applying electronic health information to their health concerns, employing a 5-point Likert scale ranging from “strongly agree” to “strongly disagree” for each item (20). Among current digital health screening tools, eHEALS is often used in published research (26–29). However, it was not intended for use in clinical settings and is centered on electronic health information literacy. As such, while eHEALS does screen for aspects of digital literacy, it does not screen for Internet access, device access, or the skills needed to engage in digital health care. The DHLS is a 3 question measure that also uses Likert scale response options to evaluate confidence in the foundational digital skills necessary for accessing healthcare services (like using applications and programs on cell phones or computers, setting up video chats, and independently solving basic technical issues) (20). Because of its length, the DHLS does not comprehensively assess digital health needs. The questions are centered around digital literacy; Internet and device access are not addressed. The Veterans Health Administration’s Assessing Circumstances & Offering Resources for Needs (ACORN) is an 11 question tool targeting unmet social needs among Veterans, including digital requirements for accessing healthcare online (22). While the VA ACORN does screen for Internet and device access, it does not include digital literacy. The Health Information National Trends Survey (HINTS), conducted by the National Cancer Institute, assesses public knowledge, attitudes, and behaviors regarding health information, specifically addressing Internet use for health-related purposes. HINTS includes questions on access to health technology and focuses on digital literacy and privacy concerns but assumes Internet and device access. The American Community Survey (ACS) (conducted by the Census) includes questions on digital health such as: whether anyone in the household owns specific electronic devices, whether anyone in the household has access to the internet, and the type of Internet available.

Even the most well-cited tools are not optimal for real-world implementation in clinical settings. Moreover, few screening questions are available in non-English languages; given the well-documented disparities in use of digital health tools by populations with LEP, the lack of non-English screening questions highlights a critical gap. To expand on these observations on real-world use, our collaborative discussed patient interview we had all conducted at each of our respective sites on patient-centered approaches to address screening for risk of digital health exclusion.

## 4 Patient perspectives- *digital health questions that “speak” to patients*

Patient cognitive interviews were conducted in English and Spanish at each of our sites. During the interviews, patients were asked to answer 10–25 digital health screening questions that each health system considered adopting. We asked patients to explain their reasoning for providing each response, including any areas they found unclear. Across sites, there were some common perspectives on screening for risk of digital health exclusion and how best to ask screening questions.

*Theme 1: Digital health exclusion screening may be sensitive; should only be conducted if resources will be provided.*

Patients conveyed that being asked about Internet access could be stigmatizing. For example—specifically referencing cost and inability to pay in the question felt insensitive. An alternative option was to ask about access to “affordable Internet” instead:

“It’s a very big open wound for a lot of people right now because the cost of the Internet and the cost to connect devices vastly differs.”

Some patients reported that screening should only be performed if there were meaningful resources to improve access and/or literacy barriers. If health systems move forward with screening, they must be prepared to offer concrete resources (i.e., more than a referral number to call for assistance).

“What can we do to help you. We’re not just going to give you a number. You do it, nothing ever happens. You know what I mean?”

*Theme 2: Device and Internet access questions need to be applicable to a wider variety of scenarios and preferences.*

Current screening does not distinguish between access to different types of devices. However, participants reported comfort levels that varied with different devices suggesting a need to specifically screen for access to a preferred digital device.

“I like my iPad because it is bigger. I don’t have to use my glasses. It has everything that a telephone has. The only thing is I don’t know how to dial a number on it… I would consider it to be a smartphone.”

Patients described various locations in which they accessed the Internet. Aside from internet at home, respondents described accessing the Internet at public libraries and other public locations like fast-food restaurants. This points to the need for nuanced questions that ascertain not only internet access, but the location, cost, and reliability of access, which impacts applicability for private health interactions and communications.

“I have used McDonald’s Internet, but they only let you do certain things… and sometimes it has no access, so I turn my own data back on. Sometimes I go over my daily limit on my billing cycle, so I slow down, and I try to ensure that I don’t go over on daily usage.”

*Theme 3: Digital screening questions have been poorly written for those with limited literacy in all forms (general literacy, health literacy, digital literacy).*

Conversations with patients at our various sites suggested that existing screening questions may not be written at an appropriate literacy level. At a general literacy level, we observed that many patients preferred shorter question stems and fewer response options. Likert-scale response options in particular caused confusion:

“So you strongly agree, or you don’t agree, basically, that’s it. Either they know it, or they don’t know it, right.”

Unfortunately, many screening questions require a baseline level of digital knowledge. Many questions include words like “devices,” “Internet-enabled,” or “smartphone,” which have unclear meanings to some patients. Several patients reported a belief that a “smartphone” was a fancy, expensive phone (e.g., iPhone) which was opposed to a regular cellphone (e.g., a non-name brand smartphone).

“I don’t know what to [say]. This is a ‘regular’ cellphone. It’s not Apple or anything…”

The names of specific applications (e.g., Zoom) or search engines (e.g., Bing) could also be problematic. For example, the DHLS screening tool includes the statement, “I can use applications/programs (like Zoom) on my cell phone, computer, or another electronic device (without asking for help from someone else).” Introducing a brand name unfamiliar to a patient creates even more confusion.

“What do you mean by device?… I don’t [know] what you mean by that. You need to be specific with me.”

“For search engine, if you say Google I know what you mean but if you ask about Bing, I don’t know what that is. What do you mean by that?”

*Theme 4: Patients with limited digital literacy may need nuanced, detailed help that may not be easily addressed.*

As patients reflected on screening questions that asked how much assistance they needed to troubleshoot technical challenges, they described complex barriers that were not reflected in screening questions and inadequately addressed by help desks or resources they had been provided. Specifically, patients reported repeatedly seeking detailed, step-by-step assistance from family and friends, or spending prolonged periods attempting to resolve technical difficulties. Notably, though we did not explicitly screen for social support, its importance in mitigating digital literacy challenges spontaneously came up as reflected in these quotes.

“I’ll ask my sister for help. She’ll tell me that you have to close tab to move on or follow protocol but where does it say that (on the webpage), and she says it doesn’t … instructions are not clear.”

“I try to call the company directly to ask for help … and I meddle through it and sometimes I can’t like with password issues or open too many tabs and couldn’t get back to where I should’ve been… I’ll try to do it myself for a couple of hours.”

## 5 Conclusion

Improving digital healthcare access is a matter of improving health equity, and addressing digital inclusion as a social driver of health is a critical first step forward. We hope the lessons learned from our collaborative’s conversations, review of the literature, and engagement of stakeholders provide a starting point for health systems that are developing or modifying their approaches to screening for risk of digital health exclusion.

There are several steps for digital health equity advocates: *Researchers* can advance this work by designing and evaluating digital inclusion screening questions that can be usable in real-world clinical care. Specifically, these brief questions should: connect to real-world use of digital health; be understood by and acceptable to all patients; reflect the wide variation in device access, Internet access, digital literacy; and be language inclusive. *Health system leaders* should consistently review digital exclusion data and seek collaborations with community-based organizations focused on digital inclusion that have more established resources and knowledge on how to mitigate barriers to digital inclusion. *Healthcare payors* should recognize the importance of digital inclusion and incentivize screening efforts; there is already progress in this space such as the Centers for Medicare and Medicaid Services (CMS) rule that requires Medicare Advantage plans to screen for digital health literacy (30).

Finally, all stakeholders must recognize that screening does not ultimately address the structural barriers that result in the need for screening in the first place. This will demand multi-level solutions that address upstream causes of inequities in digital health access. This entails policy level solutions like universal broadband access, provision of connected devices regardless of financial constraints, and digital literacy training available for patients in their own communities (15, 31).

## Data availability statement

The original contributions presented in the study are included in the article/supplementary material, further inquiries can be directed to the corresponding author.

## Author contributions

AH: Writing – review & editing, Writing – original draft. EK: Writing – review & editing, Writing – original draft. NK: Writing – review & editing. NL-S: Writing – review & editing. JR: Writing – review & editing. EM: Writing – review & editing. ON: Writing – review & editing. AC: Writing – review & editing, Writing – original draft.
